# Knowledge, attitude and practice on malaria prevention and sulfadoxine-pyrimethamine utilisation among pregnant women in Badagry, Lagos State, Nigeria

**DOI:** 10.5281/zenodo.10797047

**Published:** 2016-03-25

**Authors:** Josephine N. Chukwurah, Emmanuel T. Idowu, Adeniyi K. Adeneye, Oluwagbemiga O. Aina, Philip U. Agomo, Adetoro O. Otubanjo

**Affiliations:** 1 Department of Zoology, University of Lagos, Akoka, Lagos, Nigeria; 2 Nigerian Institute of Medical Research, Yaba, Lagos, Nigeria

## Abstract

**Background:**

Malaria in pregnancy is one of the major causes of mater nal morbidity and mortality as well as of poor pregnancy outcomes. We studied the knowledge, attitude and practices of pregnant women on malaria prevention, assessed their knowledge of sulfadoxine-pyrimethamine (SP) for intermittent preventive therapy in pregnancy (IPTp-SP), and used the outcomes to create awareness on malaria prevention with IPTp-SP.

**Materials and methods:**

A structured questionnaire on malaria prevention and SP utilisation was administer ed to 450 pregnant women attending antenatal clinics in both government and private health facilities in Badagry, Lagos State, Nigeria.

**Results:**

355 (78.8% ) of the pregnant women perceived malaria as a serious illness. Other responses by the respondents included: parasitic disease (13; 2.9%); caused by mosquito (5; 1.9%), while 77 (17%) said they did not know. The signs and symptoms of malaria mentioned included headache (109; 24.2%), weakness (77; 17.1%), fever (77; 17.1%) and body pains (44; 10%). 174 (58%) women indicated that they would go to a hospital when having malaria, 54 (17%) indulged in self-medication, while 32 (11%) took herbs. 43 (14%) did nothing. Malaria prevention was performed by taking herbs (134; 30%); artemisinin-based combination therapy (ACT) (123; 27%); daraprim (104; 23%); blood tonic (51; 11%); paracetamol (21; 5%) and SP (17; 4%). Mosquito control was mainly carried out by the use of insecticide spray (215; 47.7%), followed by anti-mosquito coils (95; 21%). Out of the 450 pregnant women interviewed, 350 (84.5%) said that SP was for the treatment of malaria, while 69 (15.2%) said that it was for malaria prevention. Knowledge of SP was influenced by both education (*P*<0.05) and parity (*P*<0.001).

**Conclusion:**

The majority of the pregnant women had knowledge of SP but did not know that it is used for malaria prevention. Most of the respondents took malaria-preventive measures by taking herbs but preferred to go to the hospital when suspecting that they had malaria.

## 1 Introduction

Malaria is an important disease afflicting humanity and has remained a serious health problem in sub-Saharan Africa, particularly among pregnant women [1,2]. Pregnant women in sub-Saharan Africa are more likely than non-pregnant women to become infected with *Plasmodium falciparum* malaria and have a higher density of parasitaemia [2-4]. Malaria in pregnancy has been associated with a range of deleterious effects in women and their offspring [4,5]. It has been recognised as a public health priority since the beginning of the 1980s. The severity of clinical manifestations is determined by the level of immunity before pregnancy, which depends on the intensity and stability of local malaria transmission. In low- and/or unstable transmission areas, the degree of acquired immunity of women prior to pregnancy is low, and both the mother and her foetus risk the most severe consequences of infection [4]. Malaria infections in this case are symptomatic, and the current strategy is to treat every malarial infection effectively [4]. In contrast, in areas of high malaria transmission women acquire a protective immunity prior to pregnancy.

Malaria infections are generally asymptomatic, and the current control strategy is based on the prevention of infections [6]. This involves the administration of intermittent preventive treatment (IPT) of malaria in pregnancy using sulfadoxine pyrimethamine (IPTp-SP) during pregnancy, combined with the use of long-lasting insecticidal nets (LLINs) and case management [7-10]. IPTp-SP has been a key component of the focused antenatal care package for nearly a decade, reducing the burden of low birth weight (LBW) attributable to malaria in sub-Saharan Africa [8]. In most African countries, including Nigeria, SP is recommended during the second and third trimesters of pregnancy for IPT in areas of high malaria transmission [8,9].

Studies have been conducted in various endemic areas to assess the knowledge, attitude and practices of pregnant women in preventing malaria during pregnancy, particularly the use of IPTp-SP [7,9]. Studies conducted in Ekiti and Ogun States in southwest Nigeria [11,12] and in Uganda [13] showed that the use of IPT was still suboptimal. The aims of our study were to assess the knowledge, attitude and practices of pregnant women on malaria prevention, to assess their knowledge of SP for IPT in pregnancy, and to use the outcomes for the creation of awareness on malaria prevention with IPTp-SP among pregnant women or women of child-bearing age.

## 2 Materials and methods

### 2.1 Study area

The study area was Badagry local government area (LGA), a peri-urban area in Lagos State in southwestern Nigeria, lying in the tropical rainforest belt. It covers an area of 71,426 km^2^ and has a projected population of 213,438 (2010 National Population Commission). Badagry LGA has twenty wards, and the native language is Yoruba. Malaria is endemic and perennial in this area, with a peak during the rainy season (April to September). The local economy is based on agriculture and trading, and the literacy level is high.

### 2.2 Study design

The design of the study was descriptive and cross-sectional. The sample population was selected with a cluster random sampling procedure. Three communities, namely Pota, Igborosun and Ikoga Zebbe, were randomly selected from one of the wards in Badagry LGA. The questionnaire of Otubanjo *et al*. [14], was adapted, field-tested, and administered to 450 pregnant women who were attending antenatal clinics in both public and private health facilities, including a traditional birth attendant home, between June and December 2011. The facilities included Pota Health Facility, Pota, General Hospital Badagry, Unique specialist hospital, Igborosun, Ayo Olu Hospital Ikoga-Zebbe and Omowumi traditional birth home, Igborosun.

The questionnaire was used to collect information on knowledge, attitude and practices from the 450 pregnant women recruited in the study. The questionnaire was translated into the local language in order to enhance the correct understanding of the contents of the instruments so as to obtain the accurate response [14]. Background information, characteristics of respondents, health-seeking behaviour of respondents during pregnancy, and knowledge and perception of malaria and SP utilisation in malaria were also collected [14].

Informed consent was obtained from the women included in the study. Ethical clearance was obtained from the Institutional Review Board (IRB) of the Nigerian Institute of Medical Research, Yaba, Lagos. The women in this study included primigravida, secundigravida and multigravida, and were of different educational backgrounds.

The data obtained were cleaned, coded, entered and analysed using Epi Info Software (6.04) (Centre for Disease Control and Prevention, Atlanta, GA, USA). Analysis was done with Chi square tests. A *P*-value less than 0.05 was considered statistically significant.

## 3 Results

### 3.1 Characteristics of respondents

The age of the women ranged from 16 to 45 years, with a mean age of 29 ± 4.3 years. Their ethnicity was: Yoruba, 277 (62%), Egun, 100 (22%), Igbo, 62 (14%) and Hausa, 11 (2%). Occupations included traders, 160 (36%), students, 98 (22.1%), fishing, 56 (12.6%) and housewives, 53 (12%). Other occupations included civil servants, 38 (9%), unemployed 42 (8.1%), while 0.7% was engaged in farming (Table 1).

**Table 1.** Socio-demographic characteristics of the 450 pregnant women included in the study.

Variable	Number	%
**Age group (yrs)**		
16-20	28	6.3
21-25	93	20.2
26-30	184	41.2
31-35	106	23.6
36-40	35	7.8
41-45	4	0.9
**Marital status**		
Married	431	95.8
Single	18	4
Separated	1	0.2
**Religion**		
Christian	345	76.7
Islam	105	23.3
**Ethnicity**		
Hausa	11	2.4
Igbo	62	13.8
Yoruba	277	61.8
Egun	100	22
**Education**		
Primary	68	15.1
Secondary	185	41.1
Tertiary	158	35.1
Other	2	0.4
None	37	8.3
**Occupation**		
Unemployed	42	8.1
Housewife	53	11.9
Farmer	3	0.7
Trader	160	36
Fishing	56	12.6
Civil servant	38	8.6
Student	98	22.1

Educationally, 68 (15%) of respondents had primary education, 185 (41%) had secondary education, and 158 (35.1%) had post-secondary education, while 37 (8%) had no education. 345 (76%) of the respondents were Christian, while 105 (24%) were Muslims. Most of the women (95.8%) were married, 4 (5%) were single, and 1 (0.2%) was separated (Table 1).

The study also showed that 162 (36%) of the pregnant women were primigravida; 103 (22.8%) were secondigravida, while 185 (41.2%) were multigravida (3 or more pregnancies). The number of pregnancies ranged from 1 to 5, while the number of children ranged between 1 and 7, with a mean of 2. Results showed that 162 (36%) and 122 (27%) of the women have 1 or 2 children, respectively. Another 90 (20%) and 58 (13%) of pregnant women have 3 or 4 children, respectively, while 18 (4%) have 5 children. The ages of the children ranged between 1 and 23 years, with a mean of 7 years. At the time of the interview, the ages of their pregnancies ranged between 8 and 38 weeks, with a mean of 28 weeks.

### 3.2 Knowledge, signs and treatment of malaria in pregnant women

The women’s understanding of malaria showed that 355 (79%) perceived malaria as a serious illness; 13 (3%) said it was a parasitic infection. Another 5 (2%) associated it with mosquito bites, while 68 (15%) had no knowledge of malaria. The signs and symptoms of malaria mentioned by the pregnant women included: headache, 109 (24.2%), fever, 77 (17%), weakness, 77 (17%), body pains, 44 (10%), and chills 42 (9.3%). Other signs and symptoms mentioned were lack of appetite, 18 (4%), and bitter tongue, 16 (3.5%), while 14 (3.1%) mentioned vomiting and yellow eyes, respectively (Table 2). On the frequency of attacks, 162 (36%) mentioned they suffered malaria once during pregnancy, while 81 (18%) had it twice, 27 (6%) had it three, and 22 (5%) had it four times. 158 (36%) did not suffer from malaria (Table 2).

**Table 2.** Knowledge of malaria and health-seeking behaviour of pregnant women attending antenatal clinics.

Variable	Number	%
**Knowledge about malaria**		
Serious illness	355	79.0
Parasitic disease	13	3.0
Caused by mosquito bite	5	1.0
Did not know	77	17.0
**Signs of malaria**		
Body pains	44	10.0
Yellow eyes	14	3.0
Fever	77	17.0
Headache	109	24.0
Weakness	77	17.0
Lack of appetite	18	3.0
Chills	42	9.0
Bitter tongue	16	4.0
Vomiting	14	3.0
Dizziness	6	2.0
Others	27	6.0
Do not know	6	2.0
**Frequency of malaria attack/year**		
Once	162	36.0
Twice	81	18.0
Three times	27	6.0
Four times	22	5.0
Do not suffer from malaria	158	35.0
**Treatment for malaria**		
Go to hospital	174	58.0
Take herbs	32	11.0
Self-medication	54	17.0
Did nothing	43	14.0

Actions taken by women to treat malaria during pregnancy included going to the hospital, 261 (58%), taking herbs, 49 (11%), self-medication, 77 (17%) with pyrimethamine, paracetamol, SP or chloroquine injection. Another 63 (14%) did nothing (Table 2).

### 3.3 Preventive measures against malaria by pregnant women

Preventive measures taken against malaria included taking herbs 135 (30%), chloroquine 126 (27%) or pyrimetham-ine 103 (23%). Other measures included the use of blood tonic, 54 (12%), paracetamol 23 (5%) or SP, 18 (4%). Mosquito control measures included use of insecticide spray, 215 (48%), mosquito coils, 95 (21%), bush clearing 27 (6%), and 18 (4%) put mosquito screening on doors and windows. Environmental cleanliness was mentioned by 27 (6%) of the women, while use of repellents was practiced by a mere 0.3%; 59 (13%) did nothing. In addition, 9 (2%) said they did not know about preventive measures. On awareness of LLINs and its use, 356 (79%) were aware of LLINs, while 94 (21%) said they were not aware. The result showed that only 13% of those who had heard of ITNs used them. On probing further whether the various actions taken to protect themselves from mosquito bites were effective, 383 (85.1%) claimed that the actions were effective in protecting them against mosquito bites, while 67 (14.9%) said these were not.

### 3.4 Knowledge of SP by pregnant women

The majority of women, 355 (79%), had knowledge of SP. The majority 380 (84.4%) who claimed to know what SP is used for said it was for treatment of malaria; 69 (15.2%) said it was for prevention of malaria while 0.3% said it is used for the treatment of typhoid fever. SP was taken once for malaria prevention during pregnancy by 337 (75%) of the women; twice by 28 (6%) or monthly by 28 (6%), while 20 (5%) took it three times. The result showed that another 8 (2%) took SP more than three times during pregnancy but 29 (6%) claimed that SP was taken whenever they were ill. The SP was taken between week 4 - 36. Fifteen per cent of pregnant women took SP in the first trimester and up to 15 weeks of pregnancy; 33% took it in the second trimester up to 27 weeks of pregnancy, and 14% took it in the third trimester (28-36 weeks). Two per cent could not remember when they took the SP, while 37% said they did not use SP during pregnancy. Two tablets of SP were taken by 8% of pregnant women while 92% took three tablets through self-medication. No explanation was given for taking 2 tablets of SP instead of 3. The SP medication was purchased from patent medicine vendors and was not prescribed by any doctor or adjusted to body weight. When probed whether the SP prevented malaria, 97% of pregnant women said it did while 3% said it did not (Table 3). Side effects experienced after taking SP included dizziness and nausea by 27.3% and 18.2%, respectively; itching of the body by 18.2%. Another 9% complained of sleeping excessively.

**Table 3.** Knowledge of SP usage among pregnant women.

Variable	Number	%
**Knowledge on SP**		
Good knowledge	345	77.0
No/limited knowledge	105	23.0
**Asked what it is used for**		
Treatment of malaria	380	84.5
Prevention	69	15.2
Typhoid treatment	1	0.3
**Usage of SP during pregnancy**		
Once during pregnancy	337	75.0
Monthly	28	6.0
Twice during pregnancy	28	6.0
Three times during pregnancy	20	5.0
>Three times during pregnancy	8	2.0
Use whenever ill	29	6.0
**Trimester when SP was used**		
First trimester (up to 15 weeks)	65	14.6
Second trimester (up to 27 weeks	149	33.0
Third trimester (28-36 weeks)	61	13.6
Cannot remember	9	1.9
Did not use SP during pregnancy	166	36.9
**Number of tablets of SP taken**		
Two tablets	37	8.1
Three tablets	413	91.9
**Asked whether it prevented malaria**		
Prevented malaria	435	96.7
Did not prevent malaria	15	3.3

### 3.5 Knowledge of SP versus parity

The relationship between parity and knowledge of SP showed that the knowledge of SP decreased with increasing parity. The majority of pregnant women 171 (38%) that had one pregnancy were knowledgeable about SP. Among those that had two pregnancies, 108 (24%) had knowledge of SP, while 72 (16%) and 59 (13%) of those that had three and four pregnancies exhibited knowledge on SP, respectively. The result also showed that only 40 (9%) of those that had five pregnancies were knowledgeable on SP. The association between parity and knowledge of SP was highly significant **(***X*2=14.1, df=4, P<0.001).

### 3.6 Knowledge of SP among pregnant women and various educational backgrounds

Knowledge about SP correlated with education level: Illiterate pregnant women (no primary education), 37 (8%); primary education, 67 (15%), and 187 (42%) had secondary education. The result showed that 159 (35%) of those with post-secondary education were knowledgeable about SP. This association was highly significant (*X ^2^*= 69.2, df=4, P=0.001) (Table 4).

**Table 4.** Knowledge of SP among pregnant women with various educational levels.

	Educational status (%)				
Knowledge of SP usage	No education	Primary education	Secondary education	Post secondary	Total
Yes	14 (4)	34 (10)	148 (43)	143 (42)	339 (100)
No	23 (21)	33 (30	39 (35)	16 (14)	111 (100)
Total	37 (8)	67 (15)	187 (42)	159 (35)	450 (100)

### 3.7 Knowledge of SP among different age groups attending health facilities

**Figure 1.**
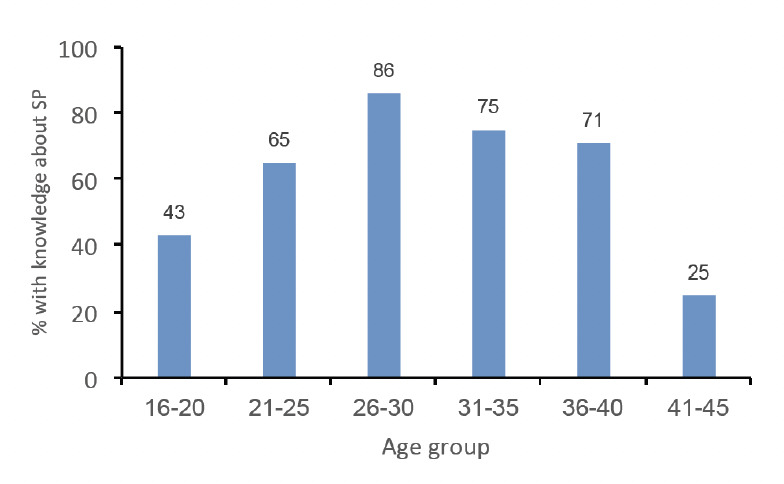
Knowledge of SP among differ ent age groups of pregnant women attending health facilities.

The result of knowledge of SP among the pregnant women of different age groups showed that 43% of pregnant women in the age bracket of 16-20 years and 65% of the age bracket 21-25 years had knowledge of SP, respectively. The result also showed that 86% and 75% from the age groups 26-30 years and 31-35 years, respectively, exhibited knowledge of SP, while 71% and 25% in the age brackets of 36-40 years and of 41-45 years, respectively, had knowledge of SP. There was no association between age and knowledge of SP (*P*>0.05) (Figure 1).

## 4 Discussion

In this cross-sectional study, community members in Badagry demonstrated a good knowledge of malaria, as is currently observed in other malaria-endemic countries. They indicated that malaria was a leading problem in the community. The knowledge of malaria exhibited by the majority of the women supports results obtained from other malaria-endemic areas [11-16]. Malaria preventive methods in Badagry were also similar to those practiced in Uganda and in other malaria-endemic countries, where, in addition to the use of insecticide sprays, doors and windows are screened, or smoked oil being poured on stagnant water to control mosquitoes [12,13,15-17].

The majority (77%) of pregnant women in Badagry LGA had knowledge of SP, and 91.9% displayed good knowledge on the correct dosage to be taken (3 tablets). This corroborates studies conducted in southwest Nigeria, where 60% of pregnant women had good knowledge of SP [11,18]. Our study showed that knowledge of SP was influenced by the educational status of the women. Pregnant women that received secondary and post-secondary education were more knowledgeable on SP than those with none or primary education. Although pregnant women between 26 and 31 years had more knowledge of SP than younger and older ones this was not significantly different.

The poor knowledge of SP as medicine for malaria prevention by pregnant women in this study corroborates an earlier study conducted in Ekiti [11], where poor knowledge of SP as a medicine for IPT was observed. The low uptake could be due to the low level of awareness and poor knowledge of the benefits of IPTp during pregnancy. This is supported by the fact that only those who have knowledge of IPT are more likely to receive at least one dose. It could also be due to lack of knowledge on the benefits of SP by health workers.

Our result is in disagreement with a study in northeast Tanzania, where pregnant women were generally aware of SP as the recommended medicine for IPT [17]. This poor knowledge of SP for IPT has implication for malaria prevention in pregnancy since pregnant women will not request or demand drugs for IPT during their antenatal clinic (ANC) visits; this hinders meeting the target of 80% of pregnant women taking IPT and sleeping under LLINs by 2010 [19,20]. Another study conducted in Tanzania reported that the majority of respondents believed that antimalarial medicines when taken during pregnancy could be harmful to the woman and her unborn child [21]. The result of a study in Uganda reported that pregnant women believed that SP is a strong medicine that weakens pregnant women, causes abortions and foetal abnormalities.

This study showed that the majority of the pregnant women visit hospitals. This clearly shows that there is an opportunity to educate these women on IPT during their ANC visits. SP is safe when given in the second and third trimester of pregnancy, but this knowledge is lacking amongst women [22]. Lack of knowledge of SP as the medicine for IPT underscores the need to create more awareness and improve specific knowledge on IPTp among women of childbearing age. Therefore, changing provider practices at ANC clinics in the delivery of IPT services, supported by community awareness campaigns to educate mothers on the importance and benefits of IPT usage, will most likely improve uptake, as demonstrated elsewhere [12,23].

## 5 Conclusions

The majority of pregnant women had knowledge of SP but did not know that it is used for malaria prevention (IPTp). Most of the respondents prevented malaria by taking herbs but would prefer to go to the hospital when suspecting they had malaria.
